# Exploiting the CRISPR/Cas9 System for Targeted Genome Mutagenesis in Petunia

**DOI:** 10.1038/srep20315

**Published:** 2016-02-03

**Authors:** Bin Zhang, Xia Yang, Chunping Yang, Mingyang Li, Yulong Guo

**Affiliations:** 1 Chongqing Engineering Research Centre for Floriculture, Key Laboratory of Horticulture Science for Southern Mountainous Regions, Ministry of Education, College of Horticulture and Landscape Architecture, Southwest University, Chongqing 400716, China

## Abstract

Recently, CRISPR/Cas9 technology has emerged as a powerful approach for targeted genome modification in eukaryotic organisms from yeast to human cell lines. Its successful application in several plant species promises enormous potential for basic and applied plant research. However, extensive studies are still needed to assess this system in other important plant species, to broaden its fields of application and to improve methods. Here we showed that the CRISPR/Cas9 system is efficient in petunia (*Petunia hybrid*), an important ornamental plant and a model for comparative research. When *PDS* was used as target gene, transgenic shoot lines with albino phenotype accounted for 55.6%–87.5% of the total regenerated T0 Basta-resistant lines. A homozygous deletion close to 1 kb in length can be readily generated and identified in the first generation. A sequential transformation strategy—introducing *Cas9* and *sgRNA* expression cassettes sequentially into petunia—can be used to make targeted mutations with short indels or chromosomal fragment deletions. Our results present a new plant species amenable to CRIPR/Cas9 technology and provide an alternative procedure for its exploitation.

During the past three decades, great efforts and achievements have been made in developing efficient tools for targeted genome modification in plants^1^,^2^. Before the CRISPR/ Cas9 system (CRISPR: clustered regularly interspaced short palindromic repeats; Cas9: CRISPR-associated protein 9) was introduced into plant research in 2013^3^,^4^,^5^,^6^,^7^,^8^,^9^,^10^, three classes of sequence-specific nucleases had been used for plant genome engineering: zinc finger nucleases (ZFNs), transcription activator-like effector nucleases (TALENs) and meganucleases^11^. Against this background, due to its versatility, design simplicity, high efficiency and low cost, the CRISPR/Cas9 technique has rapidly become a focus of attention over the past three years^1^,^2^,^12^,^13^,^14^,^15^.

The CRISPR/Cas9 system is composed of Cas9 nuclease and customizable sgRNA. The sgRNA guides Cas9 to recognize target DNA and create double strand breaks (DSBs) which trigger non-homologous end joining (NHEJ) and homologous recombination (HR) repair pathways, resulting in genome modifications^16^. Genome modification is based on the repair of DSBs^11^,^17^. Because previous reports show that the efficiency of DSB repair pathways differs between species and also cell types^1^,^17^, it is important to investigate the feasibility of the CRISPR/Cas9 system in each plant species of interest.

To date, the primary application of the CRISPR/Cas9 system in plants has been the creation of gene knock-outs^13^,^15^. It has been successfully used to induce genetic modification in plants including *Arabidopsis*^3^,^6^,^7^,^8^,^18^,^19^,^20^, *Nicotiana benthamiana*^3^,^4^,^7^,^21^, *Nicotiana tabacum*^22^, rice^5^,^6^,^7^,^8^,^9^,^10^,^23^,^24^, wheat^5^,^25^,^26^, *Zea mays*^27^, sorghum^7^, tomato^28^,^29^,^30^, potato^31^, sweet orange^32^, poplar^33^ and liverwort^34^. These reports show that CRISPR/Cas9-mediated targeted mutagenesis is efficient in plants. Analysis also suggests that the production of mutations by CRISPR/Cas9 in plants is highly specific^20^,^23^ and can be stably transmitted to subsequent generations following classic Mendelian law^18^,^19^,^20^,^23^,^24^,^28^,^35^. Although CRIPR/Cas9 has become the choice of technology for targeted genome mutagenesis in plants, its use has not been reported in ornamental plants. Because most ornamental plants are highly heterozygous and the economic importance of individual ornamental species is relatively small compared with other horticultural crops, genome-related research in ornamental plants has been retarded^36^. Affordable CRISPR/Cas9 technology is therefore poised to facilitate gene function research and genetic modification in ornamentals. Petunia is one of the most popular bedding plant species and a model system for comparative research^37^. It is a good species in which to demonstrate the application of Cas9 technology in ornamentals.

In nearly all of the studies producing stable plant mutants, the *Cas9* and *sgRNA* expression cassettes have been combined into a single binary vector. The relatively large *Cas9* expression cassette (usually larger than 5 kb) creates inconvenience in the manipulation of destination vectors. The sequential transformation strategy—transferring *Cas9* and *sgRNA* expression cassettes sequentially into plants—should therefore make the conduction of CRISPR/Cas9-mediated mutations easier. Here, we show that the CRISPR/Cas9 system is highly efficient in petunia for targeted mutagenesis. Homozygous chromosomal fragment deletions between two paired sgRNA targeted sites are readily detectable in T0 transgenic plants. The sequential transformation is an alternative strategy for Cas9-mediated genome modification in those plants amenable to *Agrobacterium*-mediated leaf-disc transformation.

## Results

### High frequency of targeted mutagenesis induced by constructs combining *pcoCas9* and an *sgRNA*

**Figure 1:**
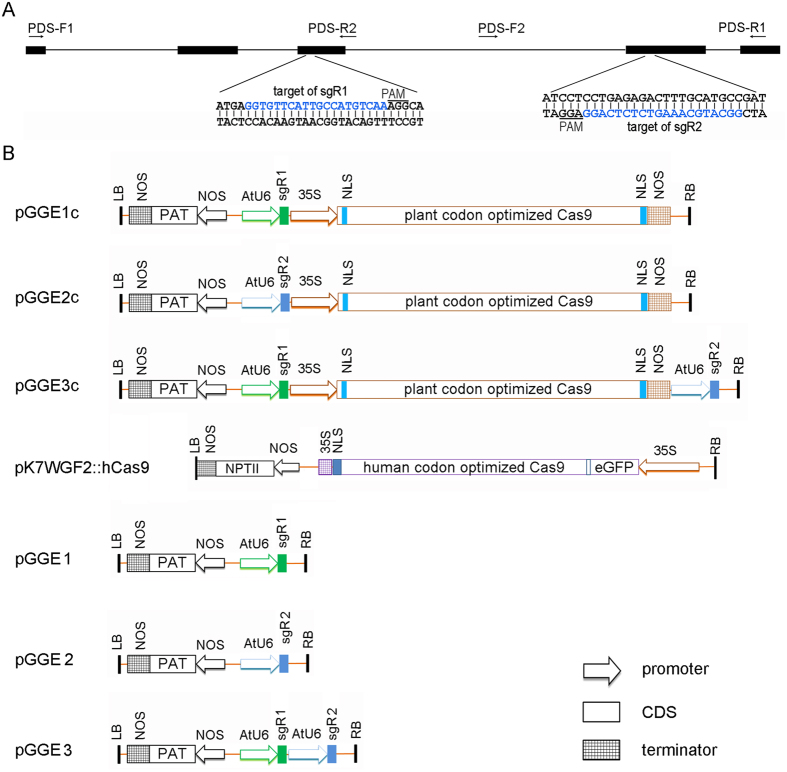
Schematic of expression cassettes used in this study. (**A**) Schematic of the petunia *PDS* gene fragment between primers PDS-F1 and PDS-R1, indicating the sgRNA target sites and sequences, the location of PCR primers. 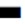 indicates exon. ▬ indicates intron. (**B**) Binary plasmids for targeted genome mutagenesis. NPTII, Neomycin phosphotransferase II; PAM, proto-spacer adjacent motif; PAT, phosphinothricin N-acetyltransferase.

Phytoene desaturase (PDS) is a key enzyme in carotenoid biosynthesis. It is required for the biosynthesis of chlorophyll. Disruption of *PDS* will cause an albino phenotype that can be easily recognized^38^. To design sgRNAs to target the petunia *PDS* gene, a fragment of *PhPDS* (GenBank ID: KP677483) genomic DNA was amplified with the primers PDS-F1 and PDS-R1 (Fig. 1A and see Supplementary Table S1 online), and the sequence was determined by Sanger sequencing. Subsequently, two sgRNAs, sgR1 and sgR2, were designed to contain guide sequences complementary to different DNA strands of *PhPDS* in the exon regions, flanking an intron of 745 bp in length (Fig. 1A).

**Figure 2:**
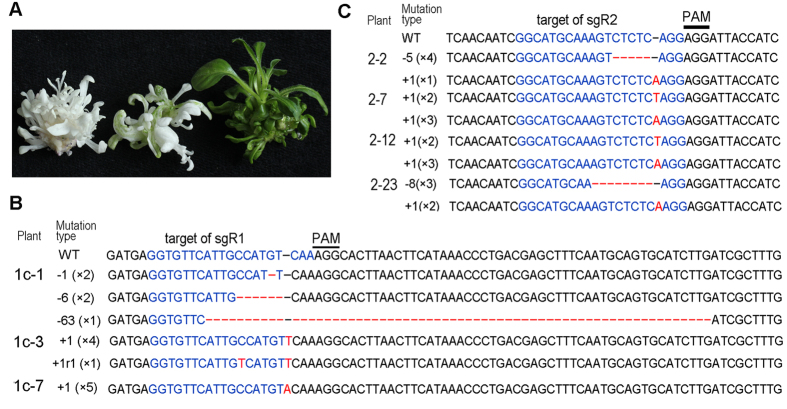
Targeted mutagenesis in petunia using the CRISPR/Cas9 system. (**A**) Bleaching phenotype of *PDS* gene mutated petunia shoots. Left, albino shoots. Middle, mosaic shoots. Right, wild type. (**B**) Targeted mutagenesis of *PDS* in pGGE1c transgenic shoots. **(C)** Targeted mutagenesis of petunia *PDS* in pGGE2 transgenic shoots using a sequential transformation approach. Deletions are denoted by red dashes. Insertions and a replacement are indicated by red letters. Figures in brackets denote the no. of detected clones with the mutation type indicated for that row. For chromatograms, see Supplementary Figures S1 and 2 online.

Albino calli began to appear at approximately four weeks after the transformation of leaf discs with the CRISPR/Cas9 constructs containing single *sgRNA*: pGGE1c and pGGE2c (Fig. 1B). All of the green and albino callus islets were excised from the leaf segments, and sub-cultured to promote shoot formation. Plantlets regenerated from the same callus islet were regarded as an independent transgenic line. PCR was performed on more than 30 randomly selected Basta-resistant plants to detect the integration of transfer DNA (T-DNA), and all of them displayed the transgene. We therefore regarded all the Basta-resistant plants as transgenic plants. Three months after transformation, a total of 214 transgenic plant lines, including albino, mosaic and green lines (Fig. 2A), were produced from approximately 300 leaf discs (200 for pGGE1c and 100 for pGGE2c) transformed with pGGE1c or pGGE2c. The proportion of transgenic lines with albino phenotype was 87.5% and 58.1% for pGGE1c and pGGE2c respectively (Table 1). Because the *Petunia hybrida* inbred line Mitchell Diploid (MD) is a diploid with two copies of each gene, the albino phenotype indicated that both copies of the target *PDS* gene had been destroyed in these transgenic seedlings.

To evaluate the types of mutation in the *PDS* locus of transgenic plants, PCR amplification was performed on three randomly selected albino plant lines transformed with pGGE1c, using the primers PDS-F1 and PDS-R2 (Fig. 1A and see Supplementary Table S1 online). PCR products were cloned and the mutation types were determined by Sanger sequencing. Plant lines were regarded as homozygous if the mutations of all of the sequenced clones (at least five clones) were the same, bi-allelic if different mutations occurred on the two copies of the target gene, or chimeric if more than three different mutations occurred on the same transgenic plant. For the three selected pGGE1c lines, one plant line was homozygous; with a 1 bp insertion located 4 bp upstream of the PAM sites (Fig. 2B, line 1c-7). One plant line (Fig. 2B, line 1c-3) was bi-allelic; one allele had a 1 bp insertion and the other allele carried a 1 bp replacement combined with a 1 bp insertion. The other transgenic line (Fig. 2B, line 1c-1) was chimeric, comprising three alleles with different deletions ranging from 1 bp to 63 bp.

### Efficient gene mutation by sequential transformation of *Cas9* and *sgRNA* expression cassettes

To determine whether targeted mutagenesis could be achieved by sequential transformation of *Cas9* and *sgRNA(s)*, the pK7WGF2::hCas9^4^ (Addgene ID, 46965) was first transferred to MD using *Agrobacterium*-mediated transformation. After selecting with 10mg/L G418, 16 independent G418 resistant plant lines were regenerated. Ten of them were subjected to PCR amplification using *hCas9*-specific primers (see supplementary Table S1 online), and eight plant lines showed the presence of the *hCas9* gene. At least 20 leaf segments from each of the eight *hCas9* transgenic lines were used as recipients for transformation with plasmid pGGE2. Thirty-six shoot lines with albino phenotype were regenerated from 50 discs of two *hCas9* plant lines (HC-40 and HC-27), and accounted for 55.6% of the total transgenic lines regenerated from these two *hCas9* plants (Table 1). This ratio was similar to that obtained using single transformation with pGGE2c containing the same sgR2 (58.1%, Table 1).

PCR amplification was performed on four randomly selected albino plant lines transformed with pGGE2, using primers PDS-F2 and PDS-R1 (Fig. 1A). Sequencing results of PCR products showed that all four plant lines were bi-allelic. In one line (line 2–2), one allele had a single adenine insertion and the other allele carried a 5 bp deletion (Fig. 2C). In a second line (line 2–23), one allele had an adenine insertion and the other allele carried an 8 bp deletion (Fig. 2C). The other two lines displayed a third type of mutation: each genome had an allele with a single adenine insertion and another with a single thymine insertion (Fig. 2C, lines 2–7 and 2–12). The results indicated that targeted mutagenesis in petunia can be achieved by sequential transformation of *Cas9* and *sgRNA* expression cassettes.

In addition, the *hCas9* transgenic plants were grown into mature plants and allowed to flower and set seed. None of these plants showed any morphological or developmental variation compared with the non-transgenic plants; this indicated that the expression of *hCas9* had no obvious detrimental effects on plant development in petunia.

### Chromosomal fragment deletion induced by CRISPR/Cas9

**Figure 3:**
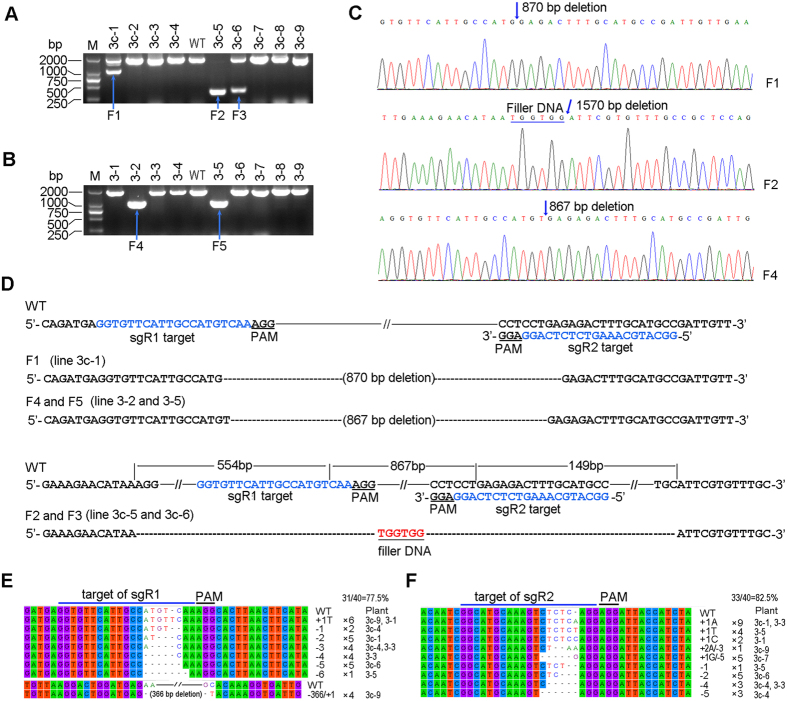
Generation of chromosomal fragment deletion by targeting two sites in the *PDS* locus of petunia. (**A,B**) Detection of deletion mutations in pGGE3c (**A**) and pGGE3 (**B**) transgenic shoots respectively. The agarose gel images indicate PCR bands amplified using primers PDS-F1 and PDS-R1. F1-5 indicates the PCR fragments with desired deletions. (**C**) Sanger sequencing chromatograms of the deletions from F1, F2 and F4 in A, B, and D. (**D**) Deletion types in five shoot lines represented by PCR fragments F1-5. (**E,F**) Other mutation types found in sgR1 (**E**) and sgR2 (**F**) target sites. Five clones per PCR product were sequenced. For chromatograms, see Supplementary Figures S3–5 online.

In order to test the capacity of the CRISPR/Cas9 system to induce chromosomal fragment deletion between two Cas9 cut sites in petunia, pGGE3c (Fig. 1B) was transferred into MD leaf segments. Sixteen albino lines were selected for PCR detection using primers PDS-F1 and PDS-R1 (Fig. 1A). Three of these lines showed a fragment close to or smaller than 1000 bp (Fig. 3A), suggesting a deletion between sgR1/Cas9 and sgR2/Cas9 cut sites. Sequencing results for the three truncated PCR products showed that they represented two classes of deletion. Fragment 1 (F1) represented an 870 bp deletion that included the 867 bp fragment between two expected Cas9 cut sites, 1 bp beyond the sgR1/Cas9 cut site and 2 bp beyond the sgR2/Cas9 cut site (Fig. 3C,D). Fragments 2 and 3 represented a 1570 bp deletion combined with a 6 bp filler DNA (TGGTGG, Fig. 3C,D), which was too short to permit a sound conclusion about its origin. Surprisingly, besides the inclusion of the fragment between the two Cas9 cut sites, the 1570 bp deletion also contained a 554 bp-long fragment extending beyond the sgR1/Cas9 cut site and a 149 bp fragment extending beyond the sgR2/Cas9 cut site; these results indicated that resection of the DSBs occurred before the breaks were re-joined. An electrophoretogram of amplicons and sequencing results demonstrated that line 3c-5 carried a homozygous deletion (Fig. 3A,D), whereas lines 3c-1 and 3c-6 contained mono-allelic deletions (Fig.3A,D).

The sequential transformation method was also tested for its ability to produce chromosomal fragment deletion in the petunia *PDS* locus. The vector pGGE3 (Fig. 1B) was transformed into HC-40 (carrying *hCas9*) leaf discs. Ten independent plant lines showing a bleaching phenotype were selected for PCR amplification using primers PDS-F1 and PDS-R1 (Fig. 1A). Two of these plant lines showed truncated PCR fragments of approximately 1000 bp in length (Fig. 3B, F4 and 5). Sequencing results showed that F4 and 5 were identical and represented a deletion between the two expected Cas9 cut sites, without additional indels (Fig. 3C,D). Taken together, the electrophoretogram and sequencing results suggested that lines 3–2 and 3–5 carried a homozygous deletion of 867 bp in the *PDS* locus. As a control experiment, when the vector pGGE3 was transformed into MD plants, no albino phenotype was observed (Table 1). The sequential transformation method can therefor be used in creating chromosomal fragment deletion in petunia.

To further identify the mutation types in pGGE3 and pGGE3c transgenic plants, eight PCR fragments from albino plants without the desired deletions were cloned and sequenced. The results confirmed that all of them carried targeted mutations. The mutations were mainly short indels, except that line 3c-9 contained a 366 bp deletion at the sgR1 targeted site (Fig. 3E,F). Mutations occurring at sgR1 and sgR2 targeted sites accounted for 77.5% and 82.5% of the total sequenced clones respectively, suggesting that both sgR1 and sgR2 possessed high efficiency.

## Discussion

In this work, for the first time, the CRISPR/Cas9 system was used to generate targeted mutagenesis in the genome of petunia plants. When constructs targeting single *PDS* sites were transformed into petunia, the proportion of bleaching phenotype occurring in T0 Basta-resistant plant lines was between 55.6% and 87.5%. These mutation frequencies were similar to those previously reported in other plants, including *Arabidopsis*(71.2%)^20^, tobacco (81.8–87.5%)^22^, tomato (48–75%)^28^, poplar (51.7%)^33^, and rice (85.4%)^39^. DSBs are mainly repaired by NHEJ in somatic cells^17^. It has been postulated that species-specific differences in NHEJ contribute significantly to the evolution of genome size^17^. Nevertheless, present data, including our results, suggest that genome size does not have a significant influence on the efficiency of targeted genome mutagenesis mediated by the CRISPR/Cas9 system. It is anticipated that Cas9 technology will promote gene function analysis and genetic improvement of highly heterozygous ornamental plants.

An advantage of the CRISPR/Cas9 system is the capacity to create targeted deletions between two Cas9 cut sites. This capacity was first verified using transient systems^3^,^21^,^22^,^25^,^40^. It was also demonstrated using transient systems that the fragment deletion efficiency was negatively correlated with the distance between two paired gRNA/Cas9 cut sites^41^. Stable transgenic plants with fragment deletions have also been obtained, but most of them involved short deletions (<250 bp)^20^,^28^,^33^,^39^,^42^. The creation of only short deletions will limit the applicability of the technique in some applications, such as the deletion of marker genes. Xie *et al.* obtained a mutant with monoallelic 727 bp deletion of *MPK5* and a mutant with a homozygous 357 bp deletion of *MPK1*^41^, and Zhou *et al.* obtained four plants with monoallelic large chromosomal fragment (~245 kb) deletions^24^. Both studies used rice as plant material. In this work, five transgenic plant lines with deletions close to 1 kb were identified from 26 albino plant lines; three of them were homozygous deletions and two were monoallelic. Because albino plants accounted for more than 45% of the single sgRNA transgenic plants in this study, and targeting one gene with two sgRNAs greatly increases mutation frequency^41^, we deduced that the proportion of plant lines with the desired deletions should be more than 8.7% of the transgenic plants; furthermore, the proportion showing homozygous deletion should be more than 5.2%. These results indicate that homozygous large deletions can be readily identified in the first generation.

In additional to the length of deletion, the efficiency of deletion may be influenced by other factors such as cell type and phase of the cell-division cycle, efficiencies of different sgRNAs, direction and base composition of the two paired sgRNAs, location and context of the target. These factors need to be addressed in future.

The homozygous deletions reached 60% (3/5) of the total deletions in this work. A previous report showed that homozygous mutations with 47–90 bp deletions were identified from stably transgenic tomato plants, and they occupied 50% (9/18) of the total deletion mutants^28^. The proportion of the homozygous deletion mutations in tomato (50%)^28^ and petunia (60%) was higher than the expected if all the deletions on the two alleles of each genome were generated independently. Ma *et al.* found in rice that the actual homozygous mutation frequency was much higher than would be expected if all the mutations were produced by the NHEJ mechanism^39^. They reasoned that the HR-based repair mechanism may be involved in the formation of homozygous mutations. However, it has been proven that HR is only a minor DSB repair pathway in somatic cells^1^. Another surprising result was that the deletions were identical between lines 3c-5 and 3c-6, and also between lines 3–2 and 3–5. A possible explanation for the identical deletions between different lines and the high proportion of homozygous deletions is that NHEJ is influenced by cell state and chromosome structure. However, the total number of reported fragment deletion events between two Cas9 cut sites in stable transformants was nevertheless very low. More data are needed to permit a valid interpretation of the deletion patterns.

Our results showed that the sequential transformation strategy worked well in petunia, with respect to both targeted mutagenesis and deletion. Although novel CRISPR/Cas9 tool-kits using Golden Gate ligation or Gibson Assembly have made the assembly of multiple sgRNA expression cassettes simple^39^,^40^,^41^,^42^, and have removed the advantage of the sequential transformation strategy in DNA fragment assembly, sequential transformation still has some other advantages, including the improvement of transformation efficiency. Because transformation efficiency declines linearly with increasing plasmid size^43^ and the expression cassette of Cas9 is usually larger than 5 kb, small sgRNA plasmids unloading Cas9 will make cloning manipulation easier. Secondly, the employment of a *Cas9* transgenic plant line in genome editing experiments can provide an identical expression level of Cas9 for genome modification. This will facilitate the comparison of different guide RNA constructs.

In addition, if the cargo capacity of a vector is limited, an sgRNA construct unloading Cas9 will also facilitate the combination of *sgRNA* with other elements such as a long HR repair template, transcriptional regulation elements and transgene expression cassettes.

There are also some disadvantages for the sequential transformation strategy. First, the resistance gene used to select *Cas9* transgenic plants will preclude the use of the same selectable marker gene in *sgRNA* transformation. However, if necessary, the resistance gene in *Cas9* plants can be destroyed or deleted using the method presented here. Another disadvantage is the difficulty of generating plants with the intended modification but carrying no foreign DNA. Because no toxic effect of Cas9 on plants has been observed, the presence of Cas9 in a plant genome may not be an acute problem in basic scientific research. Moreover, although difficult, foreign DNA can still be segregated out by outcrossing. In addition, the sequential transformation may only be applicable in certain situations. Recently, Mikami *et al.* introduced *Cas9* and *sgRNA* expression cassettes sequentially into rice calli to evaluate the frequency of mutagenesis of different constructs^44^. They found that the sequential transformation is ‘laborious’ in rice. The difference between their results and ours may originate from the fact that the transformation protocol for rice is distinct from that for petunia. In contrast, virus-mediated genome editing in plants using the CRISPR/Cas9 system has been achieved by using a sequential transformation strategy^45^,^46^. Taken together, the sequential transformation is a good alternative strategy for Cas9-mediated genome editing in those plants amenable to leaf-disc transformation.

## Material and Methods

### Plant Materials

The *Petunia hybrida* inbred line Mitchell Diploid (MD) was used in all experiments. Surface sterilized seeds were sown on ½MS semi-solid medium and grown under long-day conditions (16h light/8h dark) at 25 °C. After two weeks, seedlings were cut within the hypocotyl region and shoots were re-rooted in MS semi-solid medium. Four weeks later, young leaves were collected for *Agrobacterium*-mediated transformation.

### Vector Construction

Conventional molecular cloning procedures were used for vector construction. SgRNA expression constructs were synthesized using overlapping PCR with pUC119-AtU6::gRNA plasmid DNA (a gift from Prof. Jeen Sheen, Massachusetts General Hospital, Boston, Massachusetts, USA)^3^ as a template. PCR was carried out as described by Li *et al.*^3^. The PCR primers used in this study are listed in Supplementary Table S1 online. PCR products were cut with *Kpn*I and *Eco*RI to release the *sgRNA* expression boxes, and then inserted into the multiple cloning sites of pGreenII0229^47^ to produce pGGE1 and pGGE2 (Fig. 1B). To combine the *sgRNA* expression cassettes with the plant codon optimized Cas9 (pcoCas9) expression cassette in a single vector, the pHBT-pcoCas9^3^ plasmid DNA (a gift from Prof. Jeen Sheen) was cut with *Eco*RI and *Xho*I, and then inserted into pGGE1 and pGGE2 separately to produce pGGE1c and pGGE2c (Fig. 1B). To combine *sgR1* and *sgR2* expression cassettes into a single plasmid, we amplified the *sgR2* expression construct with PCR primers sgrF and sgrR (see Supplementary Table S1 online) to add *Xba*I and *Eco*RI to the 5′ and 3′ end of the expression cassette. After sequence verification, the *sgR2* expression cassette was released by *Xba*I and *Eco*RI, and inserted into pGGE1 to produce pGGE3 (Fig. 1B). The *sgR2* expression construct was also released by *Xba*I and *Eco*RI and inserted into pGGE1c to produce pGGE3c (Fig. 1B). All these vectors were verified by restriction enzyme cutting and sequencing.

### *Agrobacterium*-mediated transformation

The petunia transformation procedure was modified from the methods of Napoli *et al.*^48^ and Conner *et al.*^49^. In the single transformation protocol, vectors containing both *Cas9* and *sgRNA(s)* (pGGE1c, pGGE2c or pGGE3c, Fig. 1B) were used. Glufosinate-ammonium (Basta) at 4 mg/L was used to select the transgenic plants.

In the sequential transformation protocol, the plasmid pK7WGF2::hCas9^4^, obtained from Addgene (ID 46965), was initially transformed into MD plants. Subsequently, the hCas9 transgenic plants were used as recipients of sgRNA constructs pGGE2 and pGGE3 (Fig. 1B). For details of plant transformation, see Supplementary Methods online.

### Detection of mutation

One albino shoot of each line was collected, and genomic DNA was extracted using a standard CTAB method. Albino shoot DNAs of pGGE1c transgenic lines were amplified using primers designed to flank the target of sgR1 (PDS-F1 and PDS-R2, Fig. 1A). Genomic DNAs of albino pGGE2 transgenic plants were amplified using primers designed to flank the target of sgR2 (PDS-F2 and PDS-R1, Fig. 1A). The PCR products were purified using an AxyPrep PCR Clean-up kit (Axygen, Union City, CA, USA) and cloned using a pMD19-T cloning kit (Takara, Dalian, China) for sequencing.

Albino shoots of pGGE3 and pGGE3c transgenic lines were genotyped for deletion using the primers PDS-F1 and PDS-R1 (Fig.1A), which flank the targets of sgR1 and sgR2. PCR fragments close to or smaller than 1000 bp were selected, purified and sequenced. A further eight PCR fragments without distinct deletions were also cloned and sequenced.

A minimum of five clones per PCR product were sequenced using the M13F or M13R primers on an ABI3730 DNA analyser.

## Additional Information

**How to cite this article**: Zhang, B. *et al.* Exploiting the CRISPR/Cas9 System for Targeted Genome Mutagenesis in Petunia. *Sci. Rep.* **6**, 20315; doi: 10.1038/srep20315 (2016).

## Supplementary Material

Supplementary Information

## Figures and Tables

**Table 1:** Phenotypes of Basta-resistant plants transformed with CRISPR/Cas9 vectors.

Recipients	Vector ID	Vector architecture	No. of plant lines examined	No. of albino plant lines	No. of mosaic plant lines	Mutation rate (%)
MD	pGGE1c	Cas9 + sgR1	152	109	24	87.5
MD	pGGE2c	Cas9 + sgR2	62	28	8	58.1
MD	pGGE3c	Cas9 + sgR1 + sgR2	18	16	2	ND
hCas9 transgenic MD	pGGE2	sgR2	36	17	3	55.6
hCas9 transgenic MD	pGGE3	sgR1 + sgR2	11	10	1	ND
MD	pGGE3	sgR1 + sgR2	27	0	0	0

ND = not determined. MD = *Petunia hybrida* inbred line Mitchell Diploid.
