# Supplementary material for: Exploiting the CRISPR/Cas9 System for Targeted Genome Mutagenesis in Petunia
Source: Sci Rep. 2016 Feb 3;6:20315. doi: 10.1038/srep20315 (PMC4738242; doi:10.1038/srep20315)
Supplement: Supplementary Information [file srep20315-s1.pdf]

# Supplementary information

## Exploiting the CRISPR/Cas9 System for Targeted Genome Mutagenesis in Petunia

**Bin Zhang+, Xia Yang+, Chunping Yang, Mingyang Li*, Yulong Guo***

**Supplementary Table S1** PCR primers used in this study

| **Name** | **Sequence（5′→3′）** | **Comments** |
|---|---|---|
| PDS-F1 | ccgaagctcttcctgctccaa | For *PhPDS* genomic sequence |
| PDS-R1 | ggtactccgactaacttctccaact | For *PhPDS* genomic sequence |
| PDS-R2 | ggcgagataggattctaacctgaag | For *PhPDS* genomic sequence |
| PDS-F2 | ggtttctctgggttgaggacatac | For *PhPDS* genomic sequence |
| sgR1-R | cttgacatggcaatgaacaccaatcactacttcgactctag | *sgR1* synthesis |
| sgR1-F | ggtgttcattgccatgtcaagttttagagctagaaatagc | *sgR1* synthesis |
| sgR2-F | ccctgagagactttgcatgccaatcactacttcgactctag | *sgR2* synthesis |
| sgR2-R | ggcatgcaaagtctctcagggttttagagctagaaatagc | *sgR2* synthesis |
| sgrF | tctagaattgcccttaagcttcg | To add *Xba*I site to the 5′ end of *sgRNA* expression box |
| sgrR | gaattctgcagaattgcccttc | To add *Eco*RI site to the 3′ end of *sgRNA* expression box |
| hCas9-F | tgagatggctaaggtggatgactctt | Detection of *hCas9* construct |
| hCas9-R | cactcgcagaatatcactcagcagaa | Detection of *hCas9* construct |
| M13F | tgtaaaacgacggccagt | Universal primer (*sgRNA* synthesis) |
| M13R | caggaaacagctatgacc | Universal primer (*sgRNA* synthesis) |

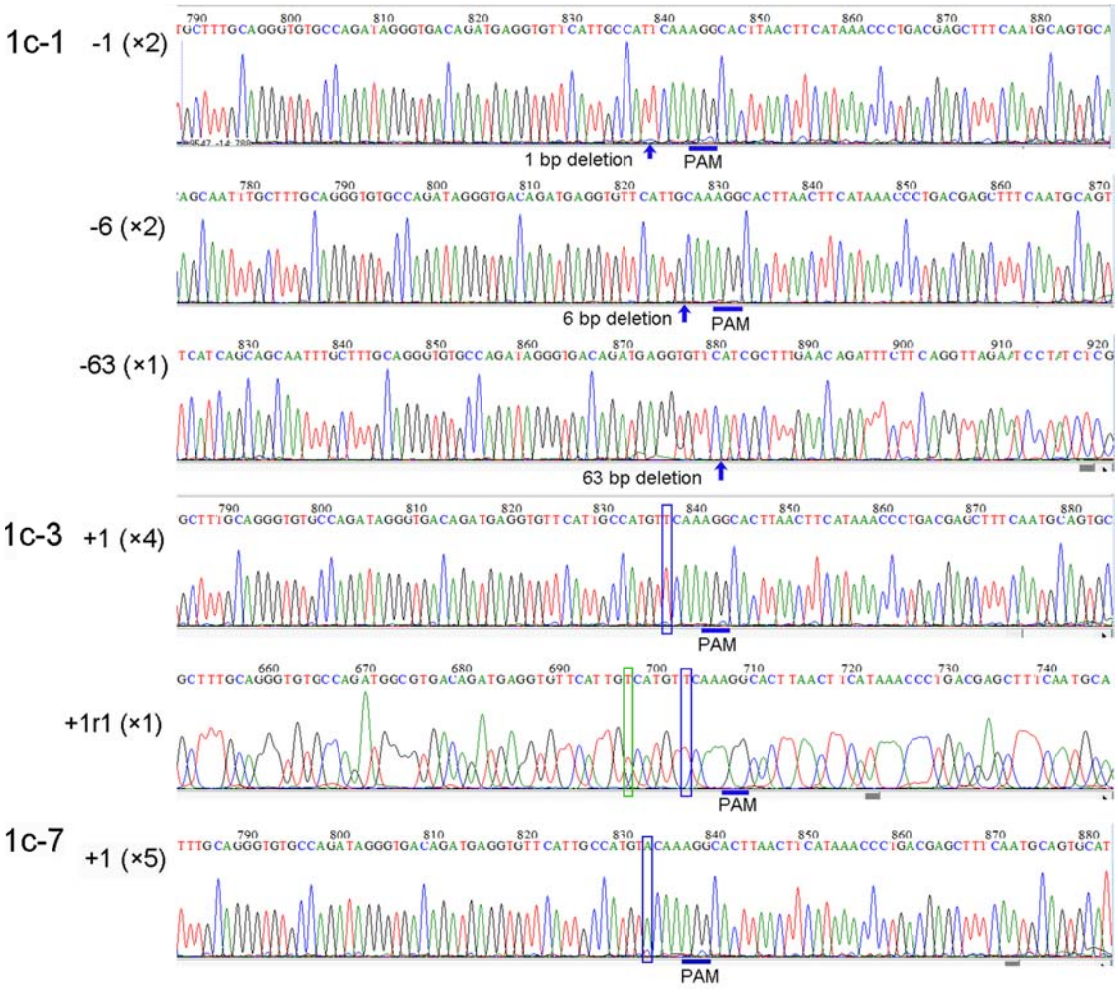


**Supplementary Figure S1.** Sanger sequencing chromatograms of PCR products from pGGE1c transgenic plants.

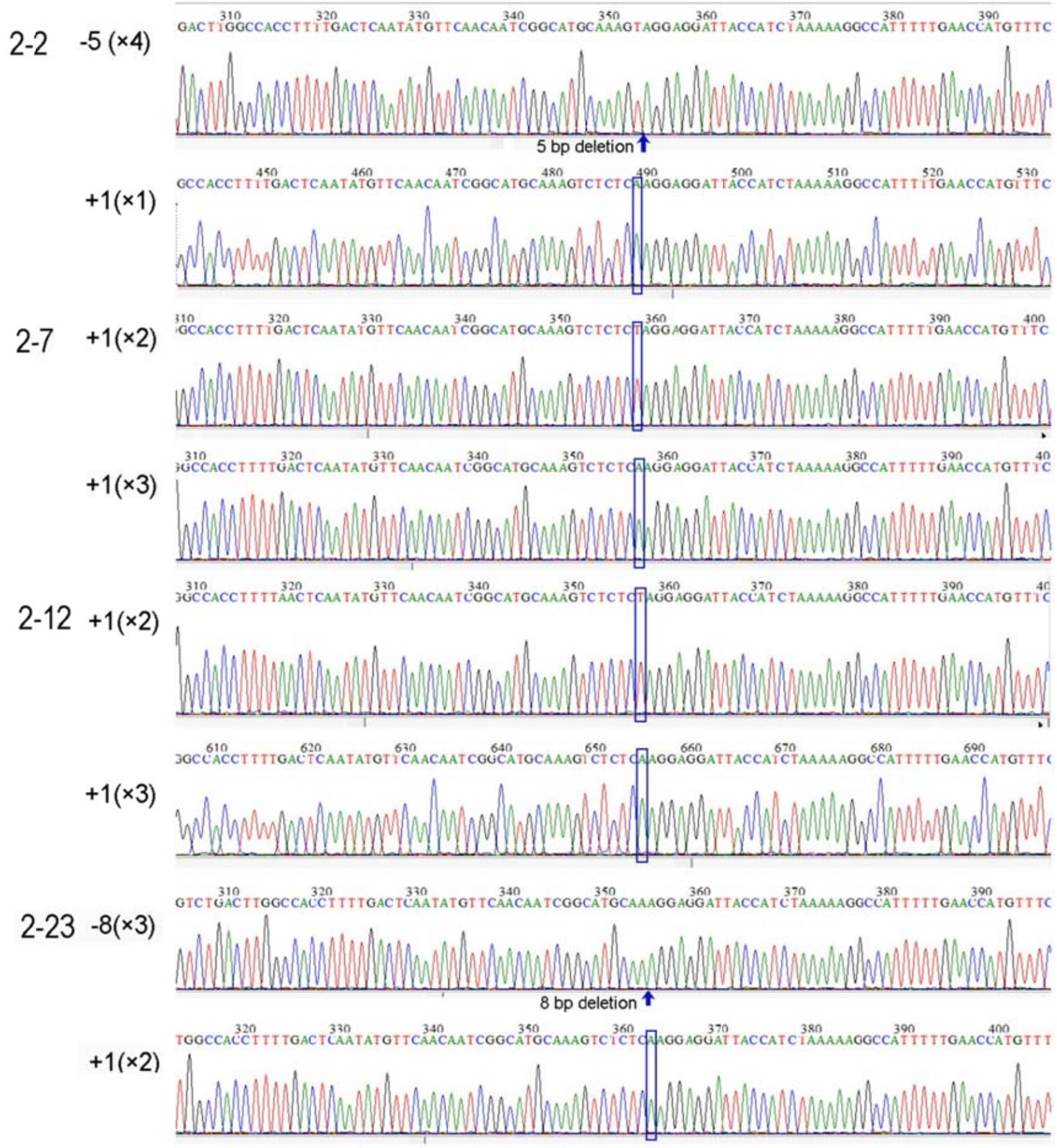


**Supplementary Figure S2.** Sanger sequencing chromatograms of PCR fragments from pGGE2 transgenic plants.

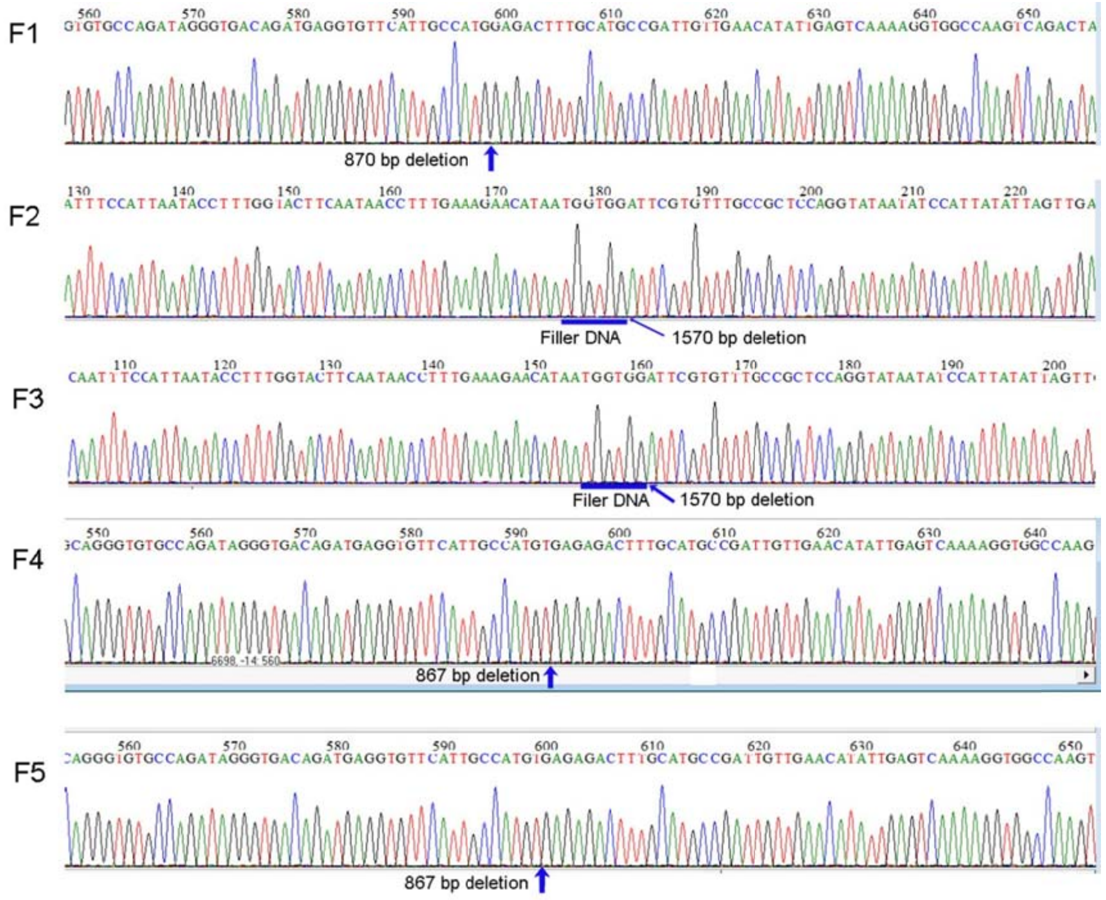


**Supplementary Figure S3.** Sanger sequencing chromatograms of PCR fragments with desired deletions from pGGE3 and pGGE3c transgenic plants.

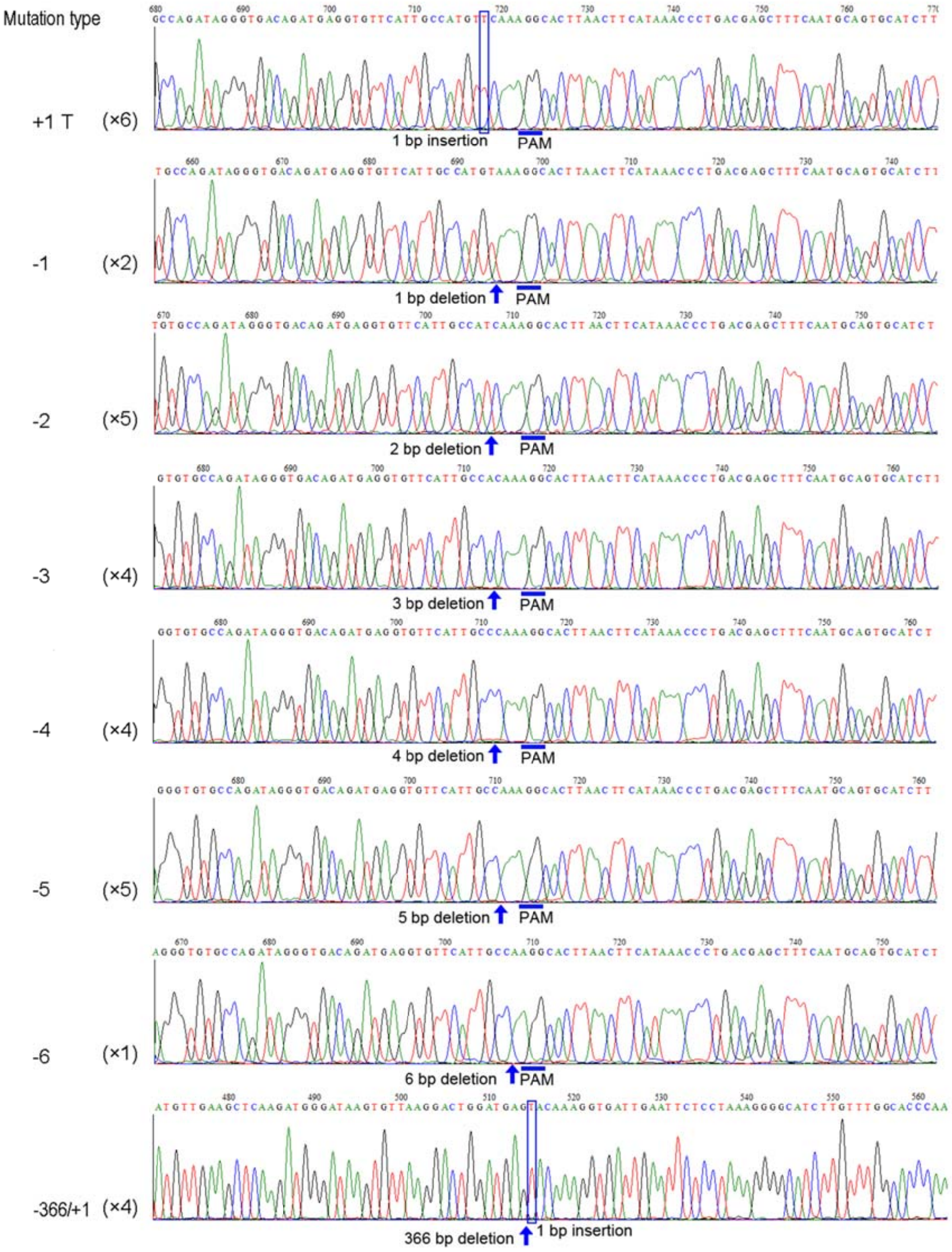


**Supplementary Figure S4.** Sanger sequencing chromatograms of PCR fragments without desired deletions from pGGE3 and pGGE3c transgenic plants, showing mutation types in sgR1targeting sites.

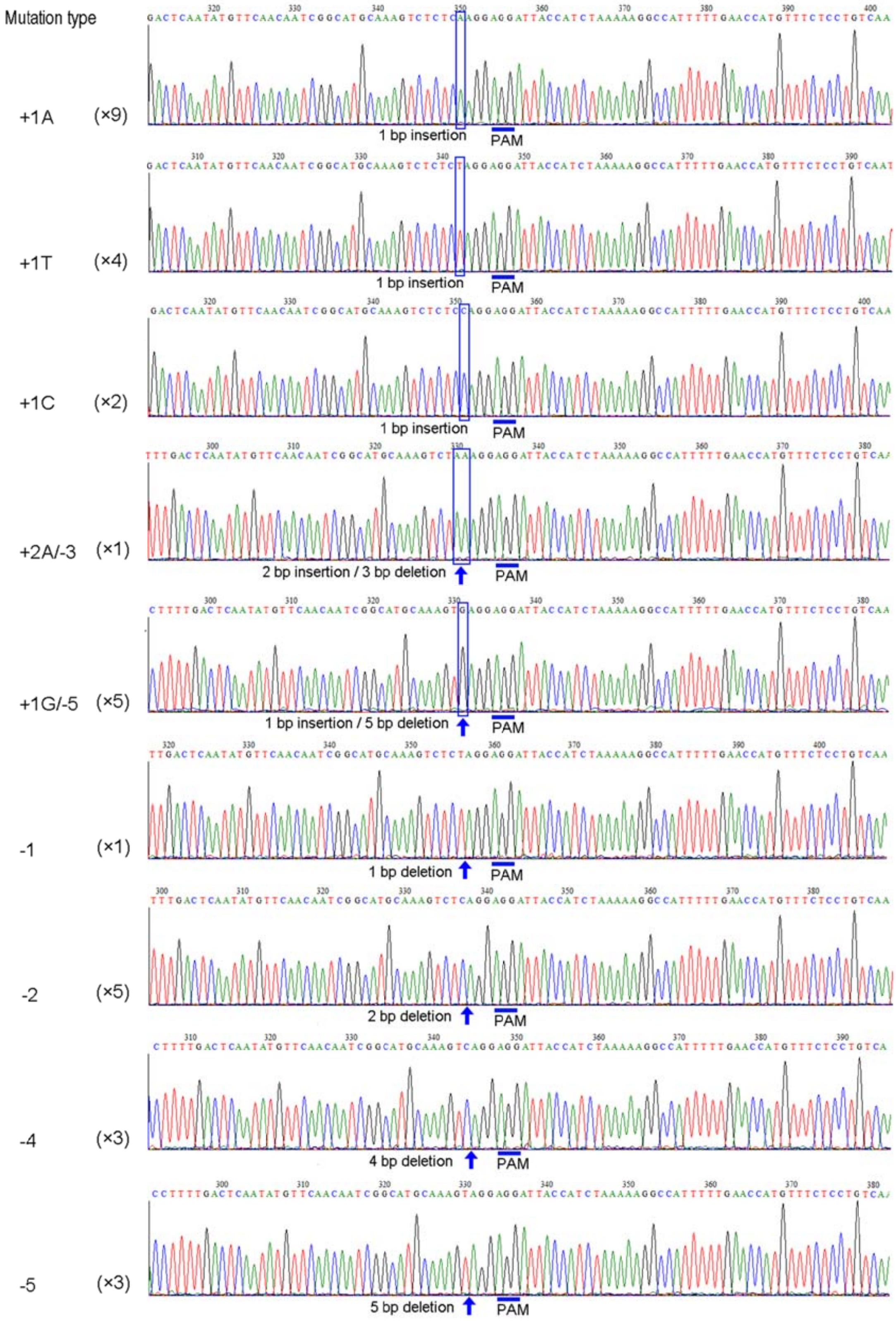


**Supplementary Figure S5.** Sanger sequencing chromatograms of PCR fragments without desired deletions from pGGE3 and pGGE3c transgenic plants, showing mutation types in sgR2 targeting sites.

**Supplementary Methods**

***Agrobacterium*-mediated transformation**

The petunia transformation procedure was modified from the methods of Napoli et al. (Napoli et al. 1990) and Conner et al. (Conner et al. 2009). Two protocols were used to transfer Cas9 and sgRNA constructs into the petunia genome. In the first protocol, we used vectors containing both Cas9 and sgRNA(s). Young leaves from aseptic seedlings of petunia MD were used as the explant sources. Leaves were cut into segments measuring about 5mm × 5 mm and pre-cultured on PS medium, consisting of MS salts, B5 vitamins, 3% sucrose, 3 mg/L BAP and 0.2 mg/L IAA, and 0.7 g/L agar at pH 5.8. After two days, leaf segments were immersed for about 5 min in a bacterial suspension of *Agrobacterium* strain GV3101 containing a selected plasmid (pGGE1c, pGGE2c or pGGE3c) (Fig. 1B). Each time the *Agrobacterium* culture was prepared for transformation, the bacterium was co-transferred with pSuper (Hellens et al. 2000) and a selected plasmid by electroporation. Single clones of GV3101 were inoculated in YEB and grown with shaking at 200 rpm at 28℃. After the presence of both pSuper and the selected plasmid was verified by PCR and the $A_{600}$ of the culture reached 0.8 to 1.5, the bacteria were collected by centrifugation and adjusted to $A_{600}$=0.2 with MB medium consisting of MS salts, B5 vitamins, 3% sucrose, 0.1% MES, and 20 μmol/L acetosyringone at pH 5.8. After inoculation, the explants were blotted with sterile filter paper and placed on new sterile filter paper on the agar surface of PS medium supplemented with 20 μmol/L acetosyringone. After co-culture at 22 ℃ for 48 h, they were transferred to PS medium supplemented with 500 mg/L carbenicillin to prevent *Agrobacterium* overgrowth and 4 mg/L glufosinate-ammonium (Basta) to select the transformed cells. The explants were sub-cultured every two weeks. When calli formed, callus islets were excised from the

leaf-segments, and sub-cultured separately. When adventitious shoots appeared, the shoots regenerated from each callus islet were considered to constitute an independently transformed line.

In the sequential transformation protocol, Cas9 and sgRNA(s) were placed in separate vectors. We first expressed human codon optimized Cas9 (hCas9) protein in MD plants. After plantlets had regenerated, the transgenic lines that had integrated hCas9 were used as recipients of sgRNA constructs. The plasmid pK7WGF2::hCas9 (Nekrasov et al. 2013), obtained from Addgene (ID 46965), was used to express hCas9 in MD plants. The transformation was conducted as described above except that 10 mg/L G418 instead of 4 mg/L glufosinate-ammonium (Basta) was used as the selective agent to select transgenic lines. Integration of the hCas9 gene in G418-resistant petunia plantlets was determined by PCR amplification using the primers hCas9F and hCas9R (Table 1) and leaf genomic DNA as a template. Leaf segments from hCas9 transgenic plantlets were used as recipients of sgRNA vectors the pGGE2 and pGGE3, and the transformation was carried out as described above.

Conner AJ, Albert NW, Deroles SC (2009) Transformation and regeneration of petunia. In: Gerats T, Strommer J (eds) Petunia: Evolutionary, Developmental and Physiological Genetics. Second Edition edn. Springer, New York, pp 395-410. doi:10.1007/978-0-387-84796-2

Hellens RP, Edwards EA, Leyland NR, Bean S, Mullineaux PM (2000) pGreen: a versatile and flexible binary Ti vector for Agrobacterium-mediated plant transformation Plant Molecular Biology 42:819-832 doi:10.1023/a:1006496308160

Napoli C, Lemieux C, Jorgensen R (1990) Introduction of a chimeric chalcone synthase gene into petunia results in reversible co-suppression of homologous genes *in trans* The Plant cell 2:279-289 doi:10.1105/tpc.2.4.279

Nekrasov V, Staskawicz B, Weigel D, Jones JD, Kamoun S (2013) Targeted mutagenesis in the model plant *Nicotiana benthamiana* using Cas9 RNA-guided endonuclease Nature biotechnology 31:691-693 doi:10.1038/nbt.2655
